# Keel bone fractures affect laying hens’ mobility, but no evidence for reciprocal effects

**DOI:** 10.1371/journal.pone.0306384

**Published:** 2024-07-05

**Authors:** Camille M. Montalcini, Michael J. Toscano, Lucy Asher, Matthew B. Petelle

**Affiliations:** 1 ZTHZ, Division of Animal Welfare, VPH Institute, University of Bern, Zollikofen, Switzerland; 2 Graduate School of Cellular and Biomedical Sciences, University of Bern, Bern, Switzerland; 3 School of Natural and Environmental Sciences, Newcastle University, Newcastle upon Tyne, United Kingdom; University of Hawai’i at Manoa, UNITED STATES

## Abstract

Keel bone fractures (KBF) are prevalent in commercial laying hens and are considered one of the greatest welfare concerns in the egg-production industry. While clear associations exist between KBF and animal mobility, suggesting that KBF impair mobility, the effect of mobility on KBF remains unclear. We combined data from three studies that assessed keel bone fracture severity through radiographs and monitored hens’ transitions between different zones of a multi-tier aviary system (the three tiers, a littered floor, and a winter garden) the week prior to radiograph. For each hen, we extracted two daily movement behaviours: the vertical distance travelled and the mean number of zones crossed within one transition; and two daily space-use behaviours: the time spent in the top tier and the unevenness of time spent across zones. We used hierarchical Bayesian continuous time dynamic modelling to estimate how a change in a behaviour predicted a later change in keel bone fracture severity, and vice versa. Increased fracture severity did not predict later changes in space-use behaviours, but it did predict changes in movement behaviours. Specifically, increased fracture severity led to decreased vertical travelled distance and a tendency to cross more zones within one transition, suggesting impaired mobility in hens with increased fracture severity. In contrast, we found no evidence that movement or space-use behaviours predict later change in fracture severity, challenging previous literature suggesting that vertical locomotion through jumping and flying may exacerbate keel bone fractures in complex three-dimensional systems due to increased risk of collisions. However, similar efforts accounting for the location of fractures on the keel could unveil the potential influence of movement and space-use behaviours in the formation and change (healing or worsening) of KBF and increase our ability to mitigate their effects.

## Introduction

Keel bone fractures (KBF) are recognized as a major welfare concern in the egg production industry [1–4]. The concern for the welfare of animals with KBF has global implications, given their high prevalence across countries and commercial strains, averaging between 24% and 63% depending on the housing system [5]. These fractures may have a detrimental impact on both egg-production [6–8] and animal welfare, with strong evidence indicating that hens with KBF feel pain for at least several weeks [9, 10] and show behavioural differences in highly motivated behaviours, including perching and nestbox use, which could indicate negative affective states [9, 11]. Furthermore, the presence or severity of KBF are positively associated with impaired mobility, such as reduced vertical locomotion [12], longer latency to fly from perches [7, 13], and increased time spent on the aviary’s top tier [14], an area with feed and water. Impaired mobility in hens with (more severe) KBF is possibly due to pain and physical impairment, as the keel is a site of muscle attachment [15] and involved in breathing [16, 17].

Although there are clear associations between KBF and spatial behaviours, including movement and space-use behaviours, with evidence suggesting that these fractures could impair mobility [12, 13], the effect of spatial behaviours on KBF remains poorly understood. Previous literature generally suggested movement throughout cage-free housing as a potential causal factor for KBF, suggesting that collisions with the housing structures [18–20] and the complex design of the system [21, 22] (including the height and presence of perches [20, 23, 24]) are contributory factors to KBF. However, recent pathological evidence suggested that collisions may not be responsible for the fractures located at the caudal tip of the bone, which account for the majority of KBF [25]. Instead, these fractures may be attributed to the internal pressure exerted during egg-laying [25, 26]. Also, in a recent review the highest average prevalence of KBF was reported in single-tier systems (63%), rather than in the more complex aviary systems (38.3%) [5]. However, it is important to note that their prevalence in aviary systems mostly relied on palpation which probably underestimated the prevalence of KBF [27] (out of the 27 observations only two did not rely on palpation and both observations were above 90%) [5]. Even if spatial behaviours were not causing new fractures, some behaviours may promote healing while others may exacerbate existing fractures (such as walking vs flying). Therefore, while keel bone fracture severity likely affects spatial behaviours it is also possible that a change in spatial behaviour could affect fracture severity. Yet, the two parts of this keel bone fracture severity − mobility association has rarely been studied in conjunction.

Despite the limited understanding of how spatial behaviours influence the existence and severity of keel bone fractures, modifications of housing to provide safer locomotion have already been suggested to reduce prevalence of KBF in cage-free systems. For instance, adding ramps in multi-tier aviaries to enable hens to move between the stacked areas by walking instead of jumping or flying, was suggested to decrease incidence of falls, collisions, and prevalence of KBF in laying hens [18, 28]. Multi-tier aviaries are especially relevant when studying KBF as they are increasingly prevalent in commercial production and the complex design of the system could exacerbate both the prevalence [21, 22] and severity [29] of these fractures and their consequences. Indeed, impaired mobility in this type of housing could increase the risk of dehydration, emaciation, and floor eggs, as individuals may be unable to access all resources across the aviary [30]. This impaired mobility may also explain why hens with fractures spent more time on the top tier [14], at least when equipped with both feed and water (as required by Swiss regulations). In 2019, Rufener et al. [14] provided the first evidence of an association between keel bone fracture severity and mobility in hens housed within multi-tier aviaries. However, whether differences in mobility precede or succeed the increase in keel bone fracture severity remains unclear, limiting our ability to develop more effective solutions for KBF.

In this study, we evaluated the potential bidirectional relationships between keel bone fracture severity (also referred to as fracture severity) and spatial behaviours of 376 commercial laying hens housed in a multi-tier aviary system. To increase robustness and sample size, we used two published datasets in addition to a newly collected dataset. All datasets assessed keel bone fracture severity through radiographs at 3–11 time points per hen and monitored hens’ transitions between five zones of the housing system (the three tiers, a littered floor, and a winter garden) the week prior to each radiograph. We extracted four spatial behaviours, including two movement and two space-use behaviours. We estimated how a change in behaviour predicted a later change in fracture severity and vice versa. Similar to Rufener et al. (2019) [14], we used the vertical travelled distance and the mean number of zones crossed within one transition as two movement behaviours, and the proportion of time spent on the top tier as a first space-use behaviour. Additionally, to address the possibility that hens may select locations other than the top tier to spend a greater portion of their daily time in response to more severe fractures, and given that this location may vary across days, we used as a second space-use behaviour an indicator of how unevenly hens used the five zones over the day.

We hypothesized that hens with increased keel bone fracture severity would reduce their activity and spend more time on the highest tier with feed and water. Therefore, we predicted that an increase in fracture severity would be followed by decreased vertical travelled distance, increased time spent in the top tier, and increased unevenness of time spent across zones. Also, we hypothesized that more transitions between the aviary stacked tiers would lead to a higher number of landings and, consequently, increased occurrence of falls and collisions. Therefore, we predicted that increased vertical travelled distance and increased number of tiers crossed within one transition (indicating longer and potentially more hazardous landings) would be followed by increased fracture severity. In other words, we predicted that a decrease in vertical travelled distance and number of tiers crossed within one transition would be followed by a decrease in fracture severity.

## Materials and methods

### Ethical note

All procedures from the three datasets were conducted in accordance with the cantonal and federal regulations for the ethical treatment of experimentally used animals, and all procedures were approved by the Bern Cantonal Veterinary Office (approval number for dataset 1: BE-31/15, dataset 2: BE-45/20, and dataset 3: BE-57/21). After data collection for the unpublished dataset (dataset3), all hens continued to live within standard production practices and thus remained in the flock. Therefore, we did not apply procedures that require anaesthesia or analgesia as animals were not killed for any of the procedures involved in this data collection. We also tried to relieve suffering. Specifically, when we observed the backpacks and tracking devices causing problems (e.g. impaired movement, wounds etc.) we removed the backpack from the animal. Also, animals were monitored daily by trained animal care staff and researchers which ensured that problems in the focal birds were early noticed and either treated or euthanized.

### Study design

We combined datasets from three experiments with similar spatial behaviours and assessments of keel fracture severity, both behaviour and fractures were assessed repeatedly on individual hens (*Gallus gallus domesticus*) throughout the laying period. All datasets were collected by the Center for Proper Housing (ZTHZ)–Dataset1, first published by Rufener et al. in 2019 [14], used an infrared tracking system. Dataset2 was collected for a previous study [31] and Dataset3 has not been published. Dataset2 and 3 followed similar protocols, employing the same low frequency tracking system validated and described in [32], and maintained comparable housing conditions and number of tracked hens per time point. Dataset3 focused on the flock of the year after Dataset2 was collected and had a different treatment for the purpose of other studies. Specifically, approximately half of the hens from Dataset2 hatched-on-farm while the other half hatched in a commercial hatchery. In Dataset3 half of the hens were relocated in a new identical pen three times throughout the laying phase while the other half stayed in their home pen during the entire laying phase. We have controlled for the treatments from Dataset2 and Dataset3 in subsequent analysis.

**Fig 1:**
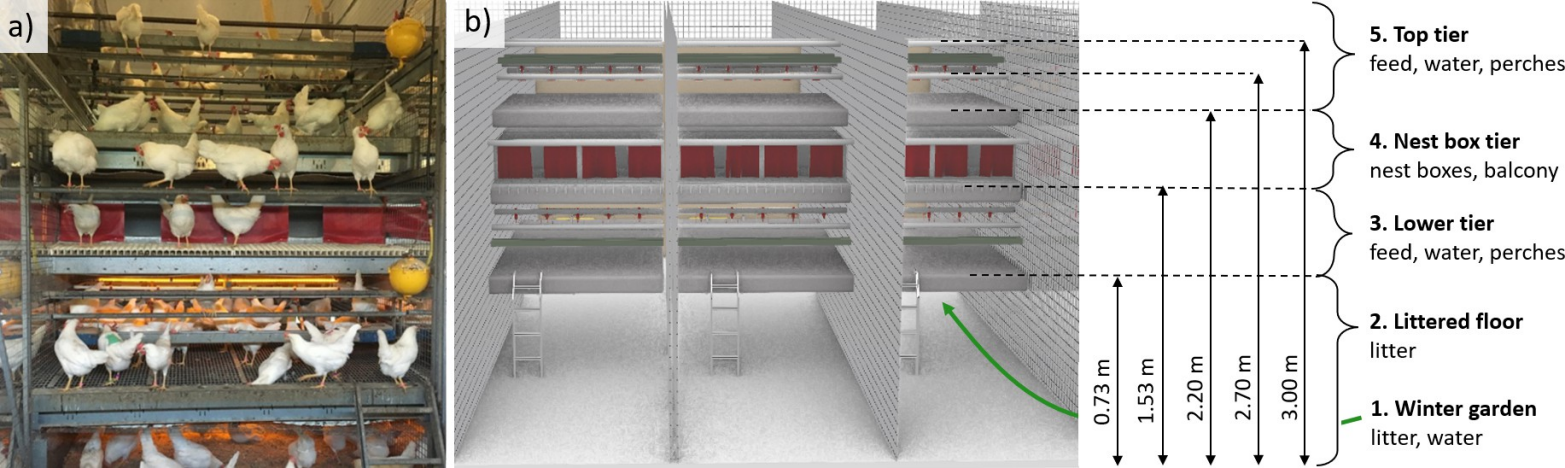
Illustration of the multi-tier aviary system. **(a)** Side view of the aviary system including a single pen with its three aviary zones (top tier, nestbox, lower tier) and the littered floor. **(b)** A simplified representation of three pens and the five zones of the housing system across which we monitored hens’ transitions.

All hens were housed over different years but in the same laying barn located at the Aviforum facilities in Switzerland where standard animal husbandry practices are used. Hens were distributed among three pens for Dataset1 (pens 4–6) and eight pens for Datasets2 and 3 (pens 3–5, pens 8–12), with a stocking density of 8.1 hens per square-meter of permanently accessible area (in each pen: 225 hens / 27.92 *m*^2^). The laying barn contained an outside covered winter garden (9.32 m^2^, accessible by pop holes from the littered floor for 6h on most days) and an aviary system illustrated in Fig 1 (Bolegg Terrace from Vencomatic, described in [18]). The laying barn is separated into 20 pens by grids (pen indoor area: 7 m length, 2.3 m width, 2.69 m height to the top tier grid floor). Each pen is composed of five zones: i-ii) a top and a lower tiers both containing feed, water, and perches, iii) a nestbox tier containing nestboxes and a balcony, iv) a littered floor, and v) a winter garden containing litter and water.

For each of the three datasets, the fracture severity was assessed at 3–11 time points per hen, and hens’ transitions between the five zones were continuously tracked for approximately a week prior each time point. On average, the week prior each time point comprised of 5.80 (SD = 0.56), 5.5 (SD = 1.77), and 6.55 (SD = 1.02) tracked days per hen for Dataset1, Dataset2, and Dataset3, respectively. We extracted two movement and two space-use behaviours (described below) per day and per hen. In subsequent analysis, we used for each time point one mean value per behaviour per hen using the available tracking data during the week prior each time point. We excluded hens that had fewer than three time points with both radiographs and behaviours (74 hens from Dataset1 and 6 hens from Dataset2, more details in S1 Text) as we analysed change over time and included individual variation in trends over time (for each behaviour and the fracture severity score, with random slope and intercept). The final dataset included a total of 376 hens, including 60 Lohmann Brown hens in Dataset1 and 153 and 163 Dekalb White hens in Dataset2 and Dataset3, respectively. Overall, the dataset comprised a total of 1,889 unique hen-timepoint, each including one value per behaviour and a fracture severity score. More precisely, Dataset1 had 593 hen-timepoint with an average of 10 time points per hen (SD = 1.73), Dataset2 had 658 hen-timepoint with an average of 4 time points per hen (SD = 0.76), and Dataset3 had 638 hen-timepoint with an average of 4 time points per hen (SD = 0.28). The youngest and oldest hens were 148 and 437 days of age respectively, at the time of being radiographed. The mean number of days between two consecutive time points for the same hen is 29.04 (SD = 9.57) for Dataset1, 63.10 (SD = 31.09) for Dataset2, and 72.81 (SD = 10.33) for Dataset3. Datasets are further detailed in S1 Text.

### Keel bone fractures

**Fig 2:**
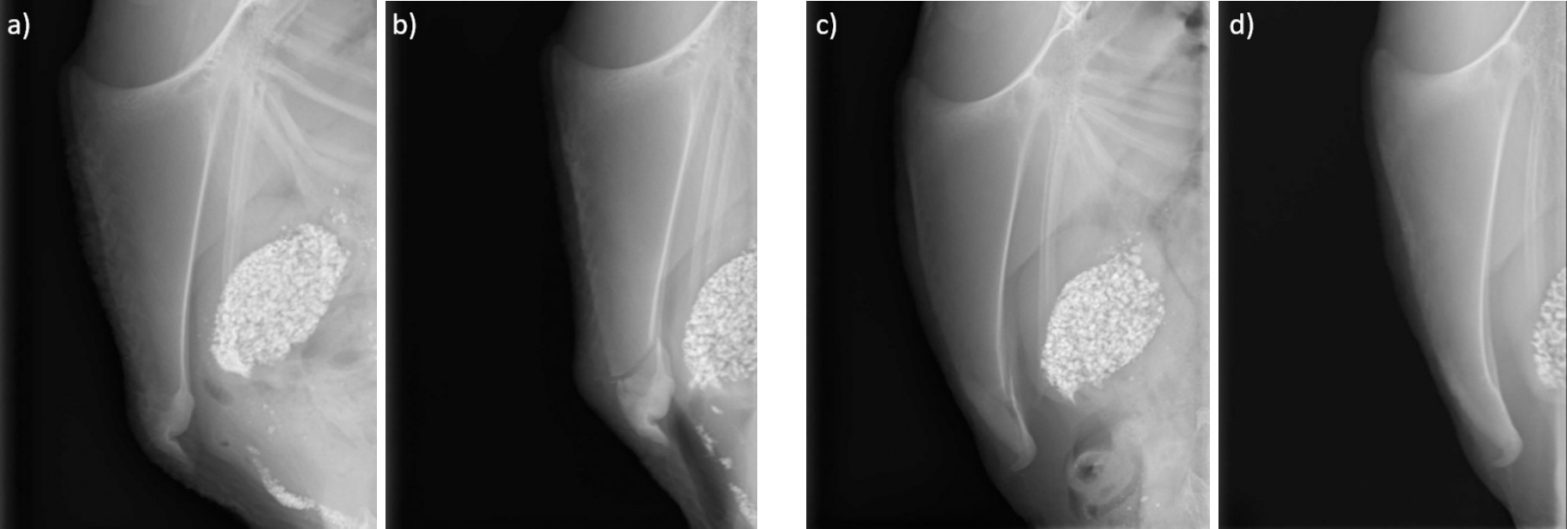
Four latero-lateral radiographs of the last two time points for two hens from Dataset2. **(a-b)** A hen which fracture severity increased in between the last two time points. **(c-d)** A hen which fracture severity decreased in between the last two time points.

The same radiograph procedure was performed in each experiment. On the first day after each tracking period, hens were radiographed to detect fractures on the keel bone, using a mobile X-ray unit previously described by Rufener et al. [33]. The hens were hung upside down from a custom-built shackle for approximately 15–30 seconds to induce immobility during the radiograph procedure. Based on the latero-lateral radiographs (illustrated in Fig 2), a fracture severity score (continuous, scaled from 0–100 as in [31, 34]) was assessed using a scoring methodology described by Rufener et al. [33]. The score is described as an indicator of the total amount of keel bone affected by any fracture. In total, two observers rated the radiographs (observer1 rated Dataset1 and observer2 rated Dataset2-3). The inter-observer reliability of the two observers was assessed in [34], while the intra-observer reliability of observer1 and observer2 were assessed in [33, 34], respectively. During assessment, observers were blind to both identity and age of the hens. The supporting information (S1 Table and S1 Fig) provide further descriptive statistics of the fracture severity score.

### Movement behaviours

Because the four indoor zones are stacked on top of each other, we used the total number of indoor zones crossed (per hour to account for different day length over experiments; mean ± SD: 14.91 ± 0.85 h) as a measure of vertical movements and hereafter referred as “vertical travelled distance”. We used the mean number of zones (tiers of the aviary and the winter garden) crossed per transition as defined by [14], hereafter referred as “mean-zone-crossed”. When the mean-zone-crossed behaviour equals to one it means that the hen did not skip zones over the day, while values higher than one indicate that the hen skipped zones while transitioning from one zone to another.

**Fig 3:**
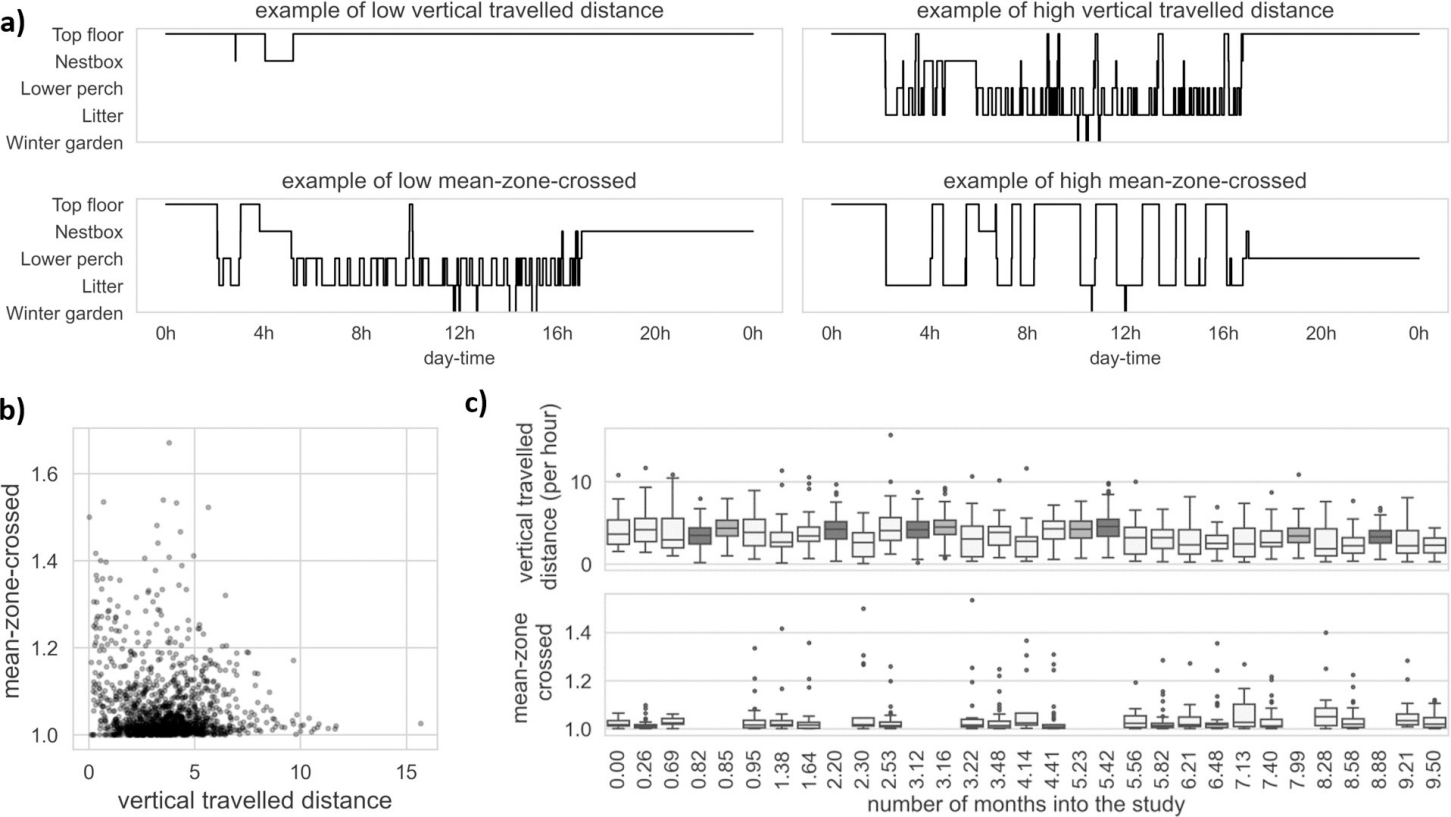
Illustration of the two movement behaviours. **(a)** Four days of the raw tracking data (transitions from one zone to another zone) chosen for visual purpose to illustrate a low and high vertical travelled distance and low and high mean-zone-crossed, as described by their respective title. **(b)** Scatter plot of the two behaviours with respect to each other, where overlapping data points are represented by darker shading. **(c)** Boxplots of the two behaviours for each time point, starting from 0 month into the study and extending beyond 9 months into the study (at time 0 hens were 148 days old). Each time point belongs to one of the three datasets, distinguished by different colours, which are best represented in the upper visual where the boxplots are generally longer. Dataset1 is highlighted in lightest grey, Dataset2 in darker grey, and Dataset3 in middle grey. Time intervals between two time points are not fixed.

To illustrate the two movement behaviours, we visually displayed four hen-days of the raw tracking data (i.e., transitions from one zone to another) with either "low" or "high" vertical travelled distance, or "low" or "high" mean-zone-crossed (Fig 3A). We also illustrated the relationship between the two behaviours in Fig 3B and showed descriptive statistics of the behaviours for each time point and dataset in chronological order in Fig 3C, where Dataset1 is represented in the lightest grey, Dataset2 in darker grey, and Dataset3 in middle grey. S1 Table provide the mean and standard deviation of the movement behaviours for each dataset.

### Space-use behaviours

Results from previous studies in the same aviary system suggested that hens with increased fracture severity spent more time in the top tier [14], and that 34% of hens spent at least one entire day on the top tier within the first three days after the transfer to the aviary system (a value that progressively reduced to 1% after 30 days) [34]. Altogether these previous results suggest that this top tier, containing both feed and water, could be of particular welfare importance. It is possible that hens use the top tier to offset pain and/or stress in response to aversive situations such as severe KBF or the transfer to new housing (e.g., by using the perches to avoid more dominant bird [35]). Therefore, as the first space-use behaviour, we used the proportion of daily time spent on the top tier.

For the second space-use behaviour, we used a score reflecting the unevenness with which hens spent their daily time across the five zones, to account for the possibility that individuals may select alternative locations to spend their time than the top tier and that the location may vary across days. For this “Unevenness” behaviour, we contrasted the hens’ proportion of time spent in each zone (with *O*_*i*_ representing the proportion of time spent in zone i) to what would be expected from an equal usage of all surface area (with *E*_*i*_ representing the proportion of the total available surface area in the pen that belong to zone i after correcting for the proportion of hours available to access that zone during the day: *E*_winter garden_: 0.11, *E*_littered floor_ = 0.48, *E*_lower tier_ = 0.17, *E*_nestbox tier_ = 0.11, and *E*_top tier_ = 0.13): $Unevenness=\frac{1}{5}\sum_{i=1}^{5}\frac{\left|E_{i}-O_{i}\right|}{E_{i}}$. Higher Unevenness scores indicate more uneven usage of the five zones.

**Fig 4:**
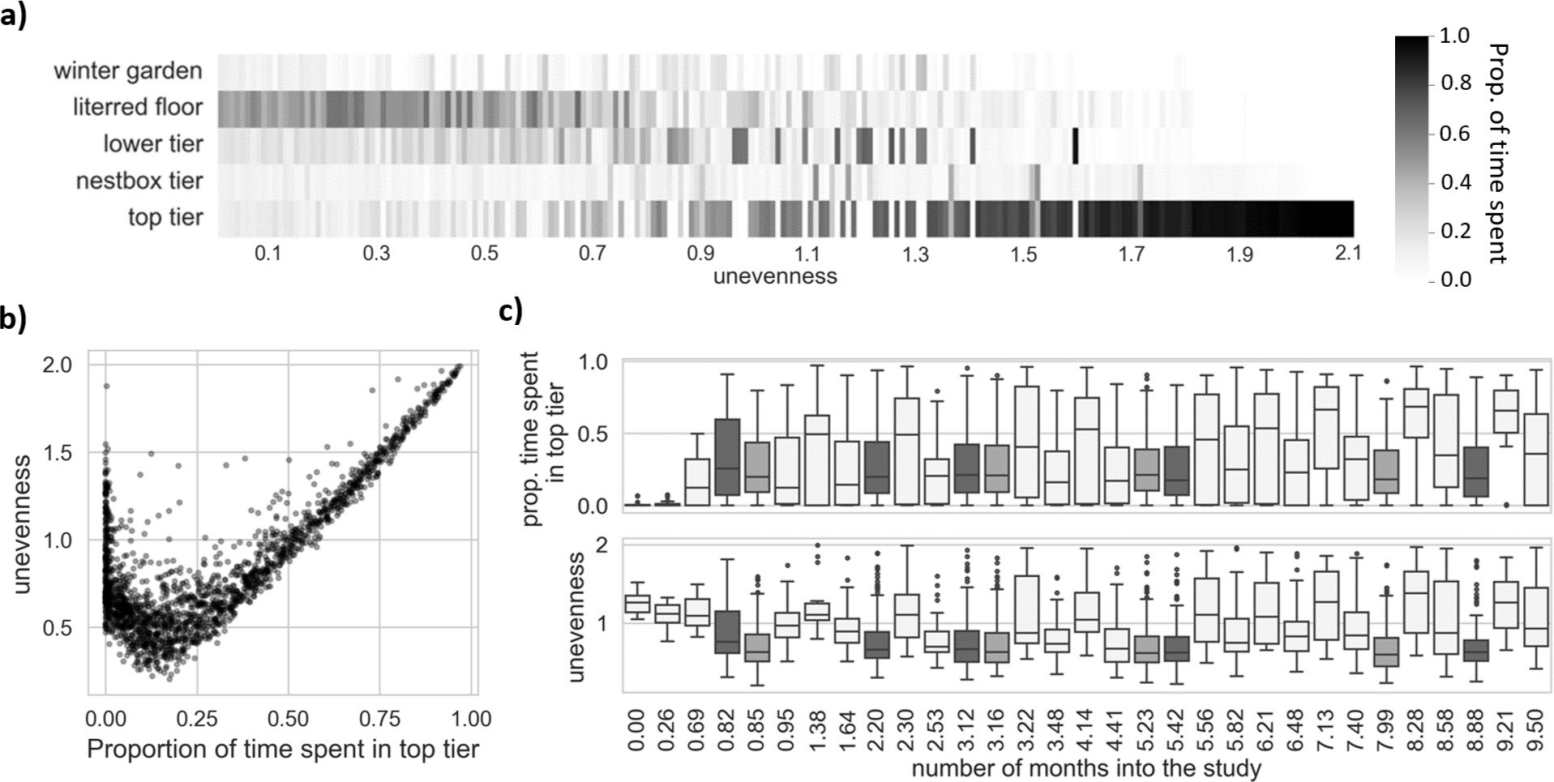
Illustration of the two space-use behaviours. **(a)** Examples of the daily proportion of time spent in each zone (darker colours for higher values) sorted by the associated daily Unevenness scores **(b)** Scatter plot of the two behaviours with respect to each other, where overlapping data points are represented by darker shading. **(c)** Boxplots of the two behaviours for each time point, starting from 0 month into the study and extending beyond 9 months into the study (at time 0 hens were 148 days old). Each time point belongs to one of the three datasets, distinguished by different colours, which are best represented in the lower visual where the boxplots are generally longer. Dataset1 is highlighted in lightest grey, Dataset2 in darker grey, and Dataset3 in middle grey. Time intervals between two time points are not fixed.

To illustrate the two space-use behaviours, we selected 210 random observations, equally distributed across possible values of the Unevenness behaviour (Fig 4A). Because the Unevenness behaviour is computed based on the proportion of time spent in each five zone, we illustrated these Unevenness scores with their associated proportion of time spent in each zone, where darker colours represent greater proportion of time. The relationship between the two space-use behaviours, illustrated in Fig 4B., shows that hens with a high daily Unevenness score, typically spent most of the day on the top tier. However, we can also observe in Fig 4A that some hens with relatively high Unevenness scores spent the majority of the day on the lower tier, a tier also equipped with feed and water. In Fig 4C we present descriptive statistics of the two behaviours for each time point and dataset in chronological order, with Dataset1 represented in the lightest grey, Dataset2 in darker grey, and Dataset3 in middle grey. S1 Table provide the mean and standard deviation of the space-use behaviours for each dataset.

### Statistics

We aimed to estimate how a change in each of the four spatial behaviours predicted a later change in fracture severity, and vice versa. We fitted four latent dynamic models using one hierarchical Bayesian continuous time dynamic model [36] per behaviour with the “ctsem” R package [37]. We used time intervals measured in months reflecting the number of months into the study, where at time 0 hens were 148 days old. We specified each model based on [38], where Driver and Tomasik described specification of multivariate latent process models that estimate the dynamics between two processes (here called variables) around their trends. Indeed, since it is expected that the severity of fractures increases with age [1, 5, 39] and that spatial behaviour changes with age [40], each model included smooth trends for both the fracture severity and behaviour to limit confounding effect of age.

Overall, each of the four models estimated a trend (over time) and a dynamic fluctuation around the trend, for both the fracture severity and the behaviour (further details on the trend and dynamic fluctuation are given in S2 Text). Each of the two trends included random initial intercepts and random slopes (varying by hen), as well as an estimated auto-effect term. The dynamic fluctuation are here referred to as “dynKBF”, “dynVTD”, “dynMZC”, “dynUnevenness”, and “dynPropZ5” for the fracture severity, vertical travelled distance, mean-zone-crossed, Unevenness, and proportion of time spent on the top tier, respectively. Importantly, we aimed to assess how fluctuations around the fracture severity trends (i.e., dynKBF) influence fluctuations around the behavioural trends (i.e., dynVTD, dynMZC, dynUnevenness, and dynPropZ5), and vice-versa. These two results are given by the model output called “drift matrix” (off-diagonal), also referred to as continuous-time temporal cross-effects parameters. To understand these continuous-time temporal cross-effects more intuitively, we extracted the expected cross-regression effects over time (discrete time parameters) to have an estimate of the temporal effect of the fracture severity on each spatial behaviour (and vice-versa) for specific time intervals. While continuous-time temporal effects describe how the variable is changing *at the moment*, discrete-time regression effects describe how the variable *looks* after it has changed for some specific period of time. In particular, discrete-time cross-regressions for a time interval of k represent the effect of one variable at *earlier* time *t*−*k* on another variable at *later* time *t*. To help interpreting these discrete-time cross-regression effects, we also reported the discrete-time auto-regression effects to have an estimate of the temporal effect of each variable on itself for specific time intervals.

As preprocessing steps previous to fitting the models, we created an empty observation row for every hen at the age of our earliest observation and set observed variables to NA when no observation was available, so that trends over time reflect trends over hens’ age (at time 0 hens were 148 days old). We scaled and centred the outcome variables and kept the default priors. For inference, we used a form of penalized likelihood, that is the maximum *a posteriori* estimation approach of ctsem [41]. In the models we controlled for the two treatments and the data source identity by allowing the trends to vary based on these predictors. We used as treatment reference group hens that hatched with the standard hatchery practices and were not relocated in new housings during production. We used as data source reference group the Dataset2 as these hens came from a single-strain commercial flock (contrary to Dataset1) and hens’ transitions between the zones were tracked with higher temporal resolution than Dataset3. To reliably measure the mean-zone-crossed behaviour, the tracking system should be accurately registering all transitions, even when the duration of stay in a zone is very brief. For instance, the tracking system should be able to differentiate a hen moving directly from the top tier to the litter floor from a hen that uses the intermediate zones briefly when moving from the top tier to the litter floor. Because Dataset2 and Dataset3 relied on a tracking system with reduced temporal resolution (emitters’ frequency of 1Hz) compared to Dataset1 (emitters’ frequency of 0.5Hz) that would not allow to reliably extract the mean-zone-crossed behaviour, we only used Dataset1 to fit the model with mean-zone-crossed as response variable. We verified normality of the observation residuals and plotted them against the time, the two observed variables, the predictors, and the predicted values of the six latent processes (called “etaprior” in ctsem).

We reported temporal effect estimates with 95% credible intervals and deem estimates significant when the credible interval does not include zero. If an estimate was not significant but its 90% credible interval excluded zero, we reported the estimate alongside its 90% credible intervals and noted the presence of a trend. We also reported all significant correlations between the random effects (i.e., the trends’ random initial intercept and slopes at the individual level for both the fracture severity and behaviour), as they may help further understand the mutual influence of fracture severity and behaviours.

## Results

### Movement behaviours

We found a negative **continuous-time temporal cross-effect** estimate of the dynKBF on dynVTD, meaning that a change in fracture severity led to a change in the opposite direction of the vertical travelled distance (estimate of -0.19, 95%CI [-0.36, -0.03]). More precisely, we found that when the standardized dynKBF increased by one, this is followed by a drop in the slope of the standardized dynVTD by 0.19. The continuous-time temporal effect of the dynKBF on the dynMZC was not statistically significant but tended to be positive (estimate of 0.33, 95%CI [-0.03, 0.70], 90%CI [0.05, 0.64]). This result indicates that when the standardized dynKBF increased by one, this tends to be followed by a rise in the standardized dynMZC slope by 0.33, which corresponds to a rise in the slope of the original mean-zone-crossed by 0.02 (= 0.33 * 0.07, where 0.07 is the SD of the original mean-zone-crossed). In contrast, we found that changes in either movement behaviours had no effect on subsequent level of the fracture severity. Specifically, the estimated continuous-time temporal effect of dynVTD on dynKBF was -0.07, 95%CI [-0.18, 0.03] and of dynMCZ on dynKBF was 0.07, 95%CI [-0.12, 0.25]. In summary, these continuous-time temporal cross-effect estimates indicated that an increase in fracture severity led to a decrease in vertical travelled distance and tended to be followed by more tiers crossed within a transition (i.e., mean-zone-crossed), but that a change in these movement behaviours did not lead to a change in fracture severity.

**Fig 5:**
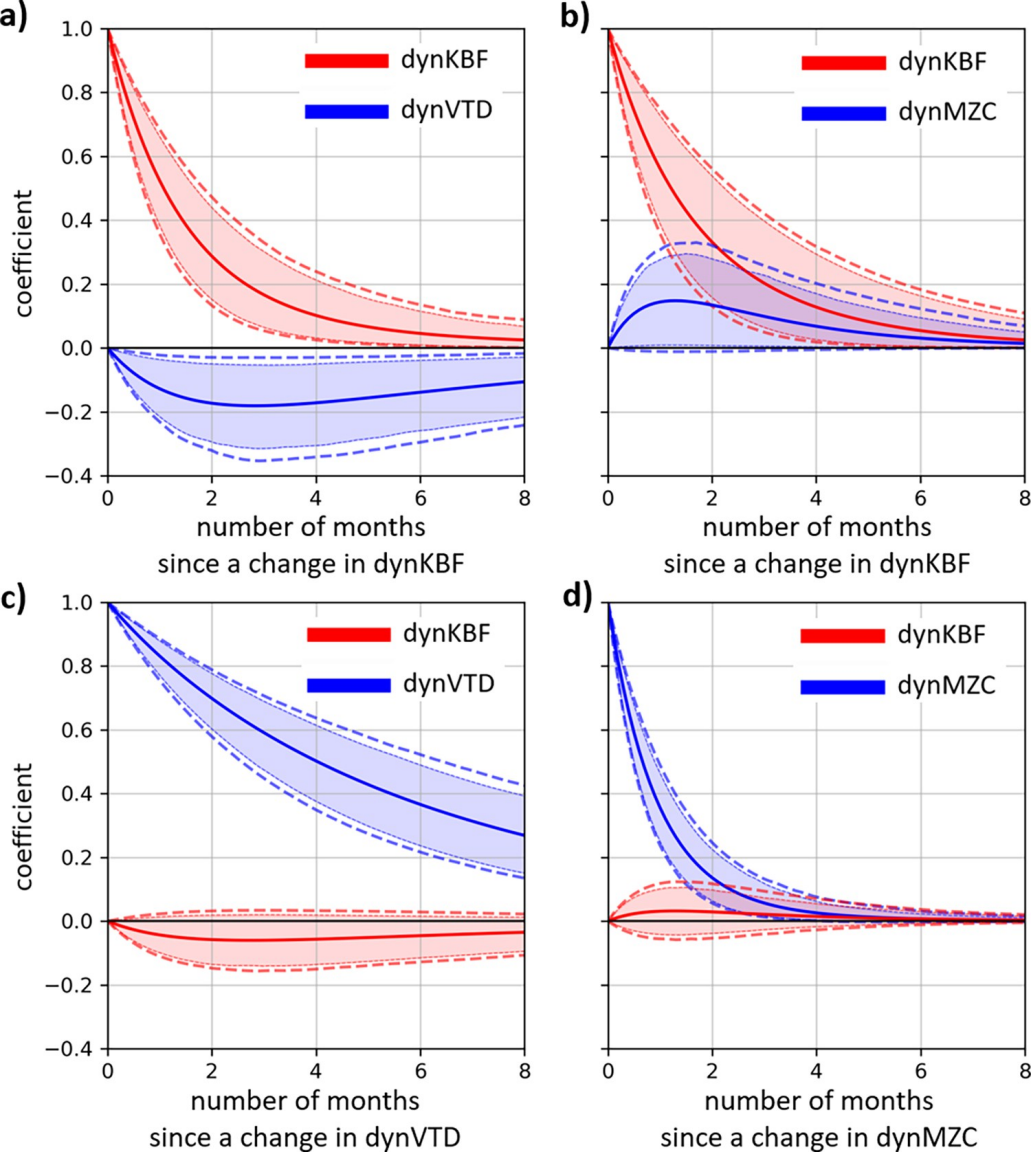
Discrete-time parameters of auto- and cross-effects of the hierarchical Bayesian continuous time dynamic models for the fracture severity and the two movement behaviours. The dynamic fluctuation of the vertical travelled distance is referred to as dynVTD and represented in **a)** and **c)** and the dynamic fluctuation of the mean-zone-crossed is referred to as dynMZC and represented in **b)** and **d)**. Mean parameter estimates for each lag are represented by the solid line and the 95% credible intervals with the most external dashed lines, so that if the value zero is not included in between these dashed lines the effect is significant. We also included the 90% credible intervals represented by the coloured area, to evaluate tendency (when value zero is not included in the dashed area).

To understand these results more intuitively we looked at the discrete-time cross-regression effects, showing how a change in the fracture severity predicts changes in the spatial behaviours after specific time intervals (Fig 5A and 5B) and vice-versa (Fig 5C and 5D). For example, at x = 3 in the Fig 5A, where the blue line equals 0.18, indicate that an increase of dynKBF by one standard deviation predicts a decrease (as the sign is negative) in the dynVTD by 0.32 three months later (i.e., 0.32 = 0.18 * 1.79, where 1.79 is the standard deviation of the vertical travelled distance from S1 Table). Because vertical travelled distance was defined as a rate (vertical travelled distance per hour), this decrease in vertical travelled distance is equivalent to a decrease of 4.8 transitions over the lighted period of a typical day (i.e., 15 hours with natural and/or artificial light). Result from the discrete-time cross-regression effects further suggest that the expected effect of fracture severity on the vertical travelled distance is greatest after approximately three months (i.e., Fig 5A, where the blue line is at its trough). It is worth mentioning that this result does not imply that any relation between the two is changing over time, but instead describes patterns of change accumulation over that time interval (Fig 5A), which can be explained by the result showing that changes in dynKBF and dynVTD are relatively persistent in time.

The discrete-time cross-regression effects indicate the extent to which these dynamic fluctuations (i.e., dynKBF, dynVTD, and dynMZC) are persistent in time, and are illustrated in red lines of the Fig 5A and 5B for dynKBF and blue lines of Fig 5C and 5D for dynVTD and dynMZC, respectively. For example, the discrete-time auto-regression effects of the dynMZC (i.e., blue line in Fig 5D) indicate that unpredictable fluctuations in mean-zone-crossed are expected to remain for a briefer period than those of fracture severity and of vertical travelled distance, implying that the dynMZC had the least predictive power on itself compared to dynKBF and dynVTD. In other words, the steepness of the blue line in Fig 5D suggests that the change in mean-zone-crossed outside that predicted from aging (i.e., change in dynMZC) are relatively rapid and thus may not be predictive of a future time point.

With regard to the trend of each variable over time, we found that vertical travelled distance diminished for the first ~3–4 months at which point the magnitude stabilized, while mean-zone-crossed followed a similar trend in the opposite direction (augmenting over time), and that the fracture severity increased through time until the end of the production period but at a slightly reduced speed over time (S2 Fig). The fracture severity trend had a substantially larger range (start until end points of the trend) than the two behavioural trends, indicative of a stronger age, or time, effect (S2 Fig).

There was one significant random effect correlation, between individual slopes of the mean-zone-crossed and individuals slopes of the fracture severity (*r* [95% CI] = 0.47 [0.15, 0.71]). This result suggests that on average, hens crossing more zones per transitions had overall greater fracture severity, supporting the idea that fracture severity is positively associated with mean-zone-crossed.

### Space-use behaviours

**Fig 6:**
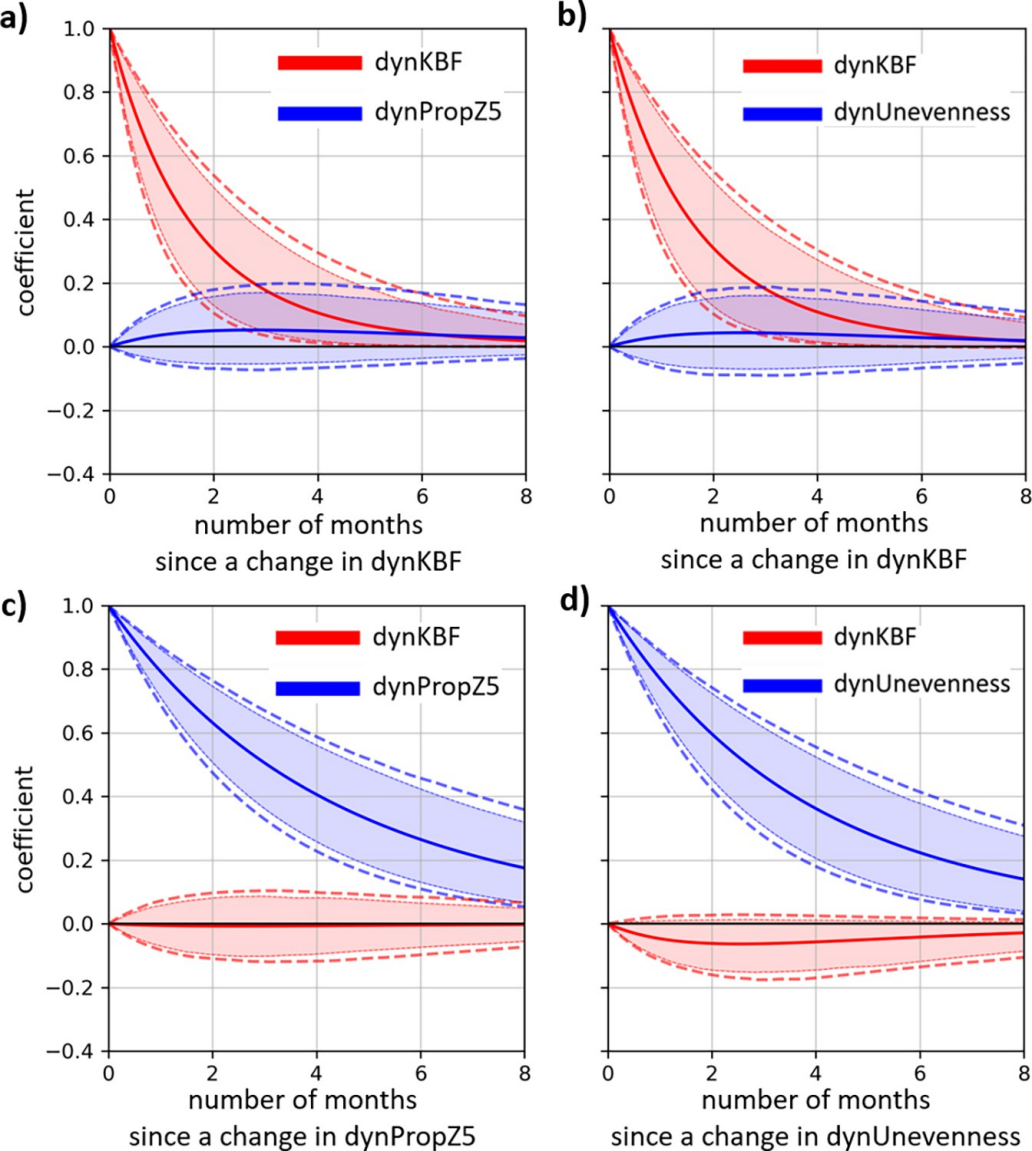
Discrete-time parameters of auto- and cross-effects of the hierarchical Bayesian continuous time dynamic models for the fracture severity and the two space-use behaviours. The dynamic fluctuation of the proportion of time spent on the top tier is referred to as dynPropZ5 and represented in **a)** and **c)**, and the dynamic fluctuation of the Unevenness behaviour is represented in **b)** and **d)**. Mean parameter estimates for each lag are represented by the solid line and the 95% credible intervals with the most external dashed lines, so that if the value zero is not included in between these dashed lines the effect is significant. We also included the 90% credible intervals represented by the coloured area, to evaluate tendency (when value zero is not included in the dashed area).

All **continuous-time temporal cross-effect** estimates were close to 0 (dynKBF on dynPropZ5: 0.06, 95%CI [-0.09, 0.19], dynKBF on dynUnevenness: 0.05, 95% CI [-0.10, 0.20], dynPropZ5 on dynKBF: -0.01, 95%CI [-0.12, 0.11], dynUnevenness on dynKBF: -0.07, 95% CI [-0.17, 0.03]). These results suggest that changes in space-use behaviours had no effect on the subsequent level of fracture severity and that changes in fracture severity had no effect on the subsequent space-use behaviours. Discrete-time parameters of auto- and cross-effects are showed in Fig 6, similar to how results from the movement behaviours were presented.

With regard to the trend of each variable over time, we found that the proportion of time spent on the top tier was increasing for the first ~3 months at which point the magnitude stabilized, and that the Unevenness was diminishing for the first ~4 months at which point the magnitude stabilized (S2 Fig). The proportion of time spent on the top tier had a substantially larger range (start until end points of the trend) than the Unevenness trend, indicative of a stronger age, or time, effect (S2 Fig).

There were two significant random effect correlations. First, individual slopes of the proportion of time spent on the top tier and of the fracture severity were positively correlated (*r* [95% CI] = 0.27 [0.07, 0.46]), indicating that hens that spent overall more time on the top tier had overall greater fracture severity, supporting the general idea that fracture severity is positively associated with the time spent on the tier. Second, individual slopes of the Unevenness behaviour and of the fracture severity were positively correlated (*r* [95% CI] = 0.31 [0.08, 0.51]), indicating that hens with a higher Unevenness asymptote (suggesting an overall more uneven usage of the zones compared to what would be expected by chance) had a higher fracture severity slope, supporting the idea that fracture severity is positively associated with Unevenness.

## Discussion

The primary objective of this study was to gain insight into whether keel bone fracture severity may be affected by spatial behaviours and whether fracture severity may, in turn, alter these behaviours. To do this, we estimated how the change in each behaviour outside that predicted from aging (i.e., change in fluctuations around behavioural trends, called: dynVTD, dynMZC, dynUnevenness, and dynPropZ5, for the vertical distance travelled, mean zones crossed, unevenness of space use, and time of the top tier, respectively) predicted a later change in fracture severity outside that predicted from aging (i.e., change in dynKBF), and vice-versa. We believe this study is the first effort to explore the bidirectional relationships between fracture severity and spatial behaviour within a single model. We found that an increase in fracture severity predicted later changes in movement but not space-use behaviours. Specifically, an increase in fracture severity was followed by a decrease in vertical travelled distance and tended to be followed by a greater mean number of zones crossed within a single transition. In contrast, we found no evidence that spatial behaviours affected fracture severity.

The decreased vertical travelled distance after an increase in fracture severity support our hypothesis that keel fractures can reduce hens’ activity. This result could be explained by the potential physical impairments in flying and walking and the perception of pain [2, 9, 10] resulting from KBF. Hens experiencing pain may be less active to facilitate the healing process or to minimize pain. While a previous study reported decreased vertical movements in hens with open fractures [12], Rufener et al. [14] did not find an association. However, since the current effort included the data from Rufener et al. [14] and we found an association, it is possible that the lack of an effect in this previous study could be attributed to a too small sample size. It is important to note that, holding all else equal, if an increase in fracture severity leads to a decreased vertical travelled distance, then those with more severe fractures will have a lower vertical travelled distance. However, due to the long-term, consistent differences in spatial behaviours observed among hens [34, 40, 42], hens are not *all equal* in behavioural expression, and therefore we cannot conclude from that result that hens with higher fracture severity also on average exhibited reduced vertical travelled distance. Instead, this result suggests that increased fracture severity induce behavioural change.

Apart from revealing new insights on the bidirectional relationships of fracture severity and hen spatial behaviour, these models also provide insights into how these effects unfold over time, which enable their comparison across studies that use different time intervals between measurements. Here, we found that the expected effect of an increase in fracture severity on the vertical travelled distance was greatest approximately three months after the change in fracture severity. In other words, a change in fracture severity was most predictive of a change in vertical travelled distance approximately three months later. The reason why we found this long-term effect is that changes in both fracture severity and behaviour were found to be relatively persistent in time. In other words, if the behaviour or fracture severity changes, we expect it to stay changed. A possible explanation for these enduring changes in the behaviour may be provided by the long-term individual consistency highlighted by previous studies in similar behaviours [34, 40]. The enduring changes in fracture severity, may be attributed to the long-lasting healing process, which can require several months to complete [1].

We also found that an increase in fracture severity tended to be followed by more zones crossed within a transition, providing further evidence of impaired vertical mobility due to fractures. By using the same dataset as in Rufener et al. [14], this result reproduced the positive association already found by that study. This result also provides new insights into the temporal sequence of changes. In contradiction to our prediction, we found that a change in fracture severity predicted a later change in mean-zone-crossed, and not the opposite. As tiers are not connected by ramps in our multi-tier aviary system, hens must transition between tiers by jumping or flying. Therefore, this result could suggest that hens with fractures attempt to minimize unnecessary stops between tiers, or, reduce the frequency of take-offs and/or landings. Prior research has showed that hens with fractures take a longer time to fly down from raised perches than those without fractures [7, 13], which could indicate an increased reluctance toward take-offs and/or landings due to heightened pain during these movements. Alternatively, hens with fractures may be physically impaired given that the keel is a site of muscle attachment [15] and involved in breathing [16, 17], which could have hindered their ability to stop in specific tiers.

The decreased vertical travelled distance in hens with increased fracture severity, along with the trend suggesting an increase in mean-zone-crossed in hens with increased fracture severity, together indicate that hens with greater fracture severity may have difficulty transitioning between tiers of this multi-tier aviary system. Yet, to access all resources within this system, hens must move vertically, as resources are distributed throughout several tiers reaching up to three meters in height. Therefore, installation of structures facilitating transitions between stacked tiers, believed to reduce the prevalence of KBF and/or facilitate simpler fracture repair (e.g., ramps that connect tiers via a walking path [18, 28]), could also improve access to all resources within the aviary for hens with KBF.

In addition to reduced vertical activity, we predicted that an increase in fracture severity would be followed by a change in space-use behaviours. We expected this due to social and resource-related motivations that would lead hens to spend more time in the top tier where they would experience fewer agonistic interactions and have access to feed and water [43, 44]. Results from the random effect correlations suggest that hens with overall greater fracture severity used the zones more unevenly and spent more time on the top tier (during the day). The latter result supports the idea that fracture severity is positively associated with time spent on the top tier, as found by a previous study [14]. However, results from the temporal effects did not provide insights into whether space-use behaviours could have affected fracture severity, and/or, as we predicted, whether fracture severity could have altered space-use behaviours. It is possible that by examining population-level effects instead of individual-level effects our methodology has masked existing temporal effects. For instance, it is possible that some hens spent more time in the top tier due to increased fracture severity, while others initially spent more time in the top tier for reasons unrelated to KBF but which subsequently led to greater fracture severity. Both conditions would support the idea of a positive association between fracture severity and the behaviour, but the presence of both effects in some hens and their absence in others could explain why we found no temporal effects.

To investigate the role of individuality in behavioural response to fracture severity, future research relying on more observations per hen and with reduced time intervals between consecutive observations, could estimate individual variation in the temporal effects (e.g., using similar models to those used in the present study [36]). Due to the limited number of observations per hen, we were unable to allow for individual variation in the temporal effects. More data would also allow to control for additional factors that could influence the effect, such as the hybrid and flock, which we could not account for in our study (as the dataset identity, although controlled for, reflect differences in the flock, the hybrid, and the tracking system).

In contrast to our result suggesting that fracture severity altered movement behaviours, we found no evidence that movement (nor space-use) behaviours altered fracture severity. Specifically, we found no evidence that a change in these behaviours was followed by a change in fracture severity (from the temporal effects results) and no evidence that the initial level of the behaviours was related to the rate of change in fracture severity (from the random effects correlations results). These results align with recent pathological evidence suggesting that collisions with infrastructure may not be responsible for the majority of the fractures (those located at the caudal tip of the bone) and instead KBF may be attributed to the internal pressure exerted during egg-laying [25, 26]. However, we did not account for the location of the fractures, which could have hidden the potential influence of spatial behaviours in the development of fractures that are not located at the tip of the bone.

Overall, our results suggest that movement behaviours in multi-tier aviaries may not cause nor exacerbate existing KBF. Yet, our movement behaviours likely relate to the amount (i.e., vertical travelled distance) and height (i.e., mean-zone-crossed per transition) of jumps/flights for which previous literature showed a considerable percentage of failed landings (9–21%) [45] that could result in KBF [18–20]. However, if KBF caused by collisions during failed landings manifest immediately after impact, rather than developing gradually (e.g., micro-fractures which would gradually weaken the overall structural integrity of the bone), our methods could not have detected it. In that scenario, to determine if collisions may cause KBF, future research with smaller time intervals between consecutive radiographs is needed.

## Conclusion

By assessing the mutual influence between keel bone fracture severity and four spatial behaviours, we found that an increase in fracture severity predicted later changes in movement behaviours, which indicated impaired mobility in hens with more severe fractures. In contrast, we found no evidence that space-use or movement behaviours affected fracture severity. However, these findings should be corroborated through studies employing shorter intervals between observations and, ideally, a greater number of observations per hen. This study focused on specific patterns of movements between areas of a multi-tier aviary system and other behaviours should be examined to more comprehensively understand whether behaviour could contribute to the maintenance and/or formation of KBF. For example, by studying the number of failed landings we could assess whether these landings exacerbate fracture severity, and whether fracture severity, in turn, can increase incidence of failed landings, resulting in an undesirable positive feedback loop. As advancements in sensor technology enable the collection of longitudinal data on behaviour and state variables, we hope this work will encourage future studies in animal welfare to explore bidirectional relationships between other state and behaviours variables.

## Supporting information

S1 Text Datasets description. (PDF)

S2 Text Trend and dynamic fluctuation description. (PDF)

S1 Fig Keel bone fracture severity scores in relation to hen’s day of age (a) and number of months into the study across datasets (b). (TIF)

S2 Fig Latent expectations conditional on the covariates, for the four trends. Because the fracture severity trend derived from each model was similar, we solely displayed the one from the model with the mean-zone-crossed.
(TIF)

S1 Table Mean and standard deviation of the four spatial behaviours and the keel bone fracture severity across datasets. (PDF)

## References

[1] Baur S, Rufener C, Toscano MJ, Geissbühler U. Radiographic Evaluation of Keel Bone Damage in Laying Hens—Morphologic and Temporal Observations in a Longitudinal Study. Front Vet Sci 2020;0:129. doi: 10.3389/fvets.2020.00129 32226794 PMC7081720

[2] Nasr MAF, Nicol CJ, Murrell JC. Do Laying Hens with Keel Bone Fractures Experience Pain? PLoS One 2012;7:e42420. doi: 10.1371/journal.pone.0042420 22927930 PMC3425496

[3] Harlander-Matauschek A, Rodenburg TB, Sandilands V, Tobalske BW, Toscano MJ. Causes of keel bone damage and their solutions in laying hens. Worlds Poult Sci J 2015;71:461–72. doi: 10.1017/S0043933915002135

[4] FAWC. Opinion on Osteoporosis and Bone Fractures in Laying Hens. London: Farm Animal Welfare Council 2010. https://www.gov.uk/government/publications/fawc-opinion-on-osteoporosis-and-bone-fractures-in-laying-hens (accessed December 18, 2023).

[5] Rufener C, Makagon MM. Keel bone fractures in laying hens: a systematic review of prevalence across age, housing systems, and strains. J Anim Sci 2020;98:S36–51. doi: 10.1093/jas/skaa145 32810250 PMC7433929

[6] Rufener C, Baur S, Stratmann A, Toscano MJ. Keel bone fractures affect egg laying performance but not egg quality in laying hens housed in a commercial aviary system. Poult Sci 2019;98:1589–600. doi: 10.3382/ps/pey544 30481360

[7] Nasr M, Murrell J, Wilkins LJ, Nicol CJ. The effect of keel fractures on egg-production parameters, mobility and behaviour in individual laying hens. Animal Welfare 2012:127–35. doi: 10.7120/096272812799129376

[8] Nasr MAF, Murrell J, Nicol CJ. The effect of keel fractures on egg production, feed and water consumption in individual laying hens. Br Poult Sci 2013;54:165–70. doi: 10.1080/00071668.2013.767437 23647178

[9] Nasr MAF, Browne WJ, Caplen G, Hothersall B, Murrell JC, Nicol CJ. Positive affective state induced by opioid analgesia in laying hens with bone fractures. Appl Anim Behav Sci 2013;147:127–31. doi: 10.1016/J.APPLANIM.2013.04.015

[10] Armstrong EA, Rufener C, Toscano MJ, Eastham JE, Guy JH, Sandilands V, et al. Keel bone fractures induce a depressive-like state in laying hens. Sci Rep 2020;10:1–14. doi: 10.1038/s41598-020-59940-1 32080271 PMC7033198

[11] Riber AB, Casey-Trott TM, Herskin MS. The Influence of Keel Bone Damage on Welfare of Laying Hens. Front Vet Sci 2018;0:6. doi: 10.3389/fvets.2018.00006 29541640 PMC5835507

[12] Rentsch AK, Rufener CB, Spadavecchia C, Stratmann A, Toscano MJ. Laying hen’s mobility is impaired by keel bone fractures and does not improve with paracetamol treatment. Appl Anim Behav Sci 2019;216:19–25. doi: 10.1016/j.applanim.2019.04.015

[13] Nasr MAF, Nicol CJ, Wilkins L, Murrell JC. The effects of two non-steroidal anti-inflammatory drugs on the mobility of laying hens with keel bone fractures. Vet Anaesth Analg 2015;42:197–204. doi: 10.1111/vaa.12175 24815351

[14] Rufener C, Abreu Y, Asher L, Berezowski JA, Maximiano Sousa F, Stratmann A, et al. Keel bone fractures are associated with individual mobility of laying hens in an aviary system. Appl Anim Behav Sci 2019;217:48–56. doi: 10.1016/j.applanim.2019.05.007

[15] Sisson S, Grossman JD, Getty R. Sisson and Grossman’s The anatomy of the domestic animals. 5th Ed Saunders 1975:2095. 10.3/JQUERY-UI.JS.

[16] Claessens LPAM. The skeletal kinematics of lung ventilation in three basal bird taxa (emu, tinamou, and guinea fowl). J Exp Zool A Ecol Genet Physiol 2009;311A:586–99. doi: 10.1002/jez.501 18942101

[17] Duncker H-R. The respiratory apparatus of birds and their locomotory and metabolic efficiency. Journal Für Ornithologie 2000;141:1–67. doi: 10.1007/BF01651772

[18] Stratmann A, Fröhlich EKF, Gebhardt-Henrich SG, Harlander-Matauschek A, Würbel H, Toscano MJ. Modification of aviary design reduces incidence of falls, collisions and keel bone damage in laying hens. Appl Anim Behav Sci 2015;165:112–23. doi: 10.1016/j.applanim.2015.01.012

[19] Stratmann A, Fröhlich EKF, Harlander-Matauschek A, Schrader L, Toscano MJ, Würbel H, et al. Soft Perches in an Aviary System Reduce Incidence of Keel Bone Damage in Laying Hens. PLoS One 2015;10:e0122568. doi: 10.1371/journal.pone.0122568 25811980 PMC4374857

[20] Sandilands V, Moinard C, Sparks NHC. Providing laying hens with perches: fulfilling behavioural needs but causing injury? Br Poult Sci 2009;50:395–406. doi: 10.1080/00071660903110844 19735008

[21] Rodenburg T, Tuyttens F, de Reu K, Herman L, Zoons J, Sonck B. Welfare assessment of laying hens in furnished cages and non-cage systems: an on-farm comparison. Animal Welfare 2008;17:363–73. doi: 10.1017/S096272860002786X

[22] Riber AB, Hinrichsen LK. Keel-bone damage and foot injuries in commercial laying hens in Denmark. Animal Welfare 2016;25:179–84. doi: 10.7120/09627286.25.2.179

[23] Pickel T, Schrader L, Scholz B. Pressure load on keel bone and foot pads in perching laying hens in relation to perch design. Poult Sci 2011;90:715–24. doi: 10.3382/ps.2010-01025 21406354

[24] Wilkins LJ, McKinstry JL, Avery NC, Knowles TG, Brown SN, Tarlton J, et al. Influence of housing system and design on bone strength and keel bone fractures in laying hens. Veterinary Record 2011;169:414–414. doi: 10.1136/vr.d4831 21862469

[25] Thøfner I, Hougen HP, Villa C, Lynnerup N, Christensen JP. Pathological characterization of keel bone fractures in laying hens does not support external trauma as the underlying cause. PLoS One 2020;15:e0229735. doi: 10.1371/journal.pone.0229735 32150551 PMC7062247

[26] Thøfner I, Shih C-C, Villa C, Christensen JP. The egg-laying process might be a contributing factor for the development of keel bone fractures in laying hens. XXIInd Congress of the World Veterinary Poultry Association, 2023, p. 228.

[27] Casey-Trott T, Heerkens JLT, Petrik M, Regmi P, Schrader L, Toscano MJ, et al. Methods for assessment of keel bone damage in poultry. Poult Sci 2015;94:2339–50. doi: 10.3382/ps/pev223 26287001

[28] Heerkens JLT, Delezie E, Ampe B, Rodenburg TB, Tuyttens FAM. Ramps and hybrid effects on keel bone and foot pad disorders in modified aviaries for laying hens. Poult Sci 2016;95:2479–88. doi: 10.3382/ps/pew157 27143777

[29] Thøfner ICN, Dahl J, Christensen JP. Keel bone fractures in Danish laying hens: Prevalence and risk factors. PLoS One 2021;16:e0256105. doi: 10.1371/journal.pone.0256105 34388183 PMC8362975

[30] Tauson R, Kjaer J, Maria GA, Cepero R, Holm K-E. The creation of a common scoring system for the integument and health of laying hens: Applied scoring of integument and health in laying hens. Final report Health from the Laywell project 2005. https://www.laywel.eu/web/pdf/deliverables%2031-33%20health.pdf (accessed December 22, 2022).

[31] Montalcini CM, Petelle MB, Toscano MJ. Commercial hatchery practices have long-lasting effects on laying hens’ spatial behaviour and health. PLoS One 2023;18:e0295560. doi: 10.1371/journal.pone.0295560 38117840 PMC10732460

[32] Montalcini CM, Voelkl B, Gómez Y, Gantner M, Toscano MJ. Evaluation of an Active LF Tracking System and Data Processing Methods for Livestock Precision Farming in the Poultry Sector. Sensors 2022;22:659. doi: 10.3390/s22020659 35062620 PMC8780220

[33] Rufener C, Baur S, Stratmann A, Toscano MJ. A Reliable Method to Assess Keel Bone Fractures in Laying Hens From Radiographs Using a Tagged Visual Analogue Scale. Front Vet Sci 2018;5:124. doi: 10.3389/fvets.2018.00124 29930948 PMC5999807

[34] Montalcini CM, Toscano MJ, Gebhardt-Henrich SG, Petelle MB. Intra-individual variation of hen movements is associated with later keel bone fractures in a quasi-commercial aviary. Sci Rep 2023;13:2377. doi: 10.1038/s41598-023-29587-9 36759525 PMC9911743

[35] Cordiner LS, Savory CJ. Use of perches and nestboxes by laying hens in relation to social status, based on examination of consistency of ranking orders and frequency of interaction. Appl Anim Behav Sci 2001;71:305–17. doi: 10.1016/s0168-1591(00)00186-6 11248380

[36] Driver CC, Voelkle MC. Hierarchical bayesian continuous time dynamic modeling. Psychol Methods 2018;23:774–99. doi: 10.1037/met0000168 29595295

[37] Driver CC, Oud JHL, Voelkle MC. Continuous Time Structural Equation Modeling with R Package ctsem. J Stat Softw 2017;77:1–35. doi: 10.18637/JSS.V077.I05

[38] Driver CC, Tomasik MJ. Formalizing Developmental Phenomena as Continuous-Time Systems: Relations Between Mathematics and Language Development. Child Dev 2022;94:1454–71. doi: 10.1111/CDEV.13990 37661359

[39] Toscano M, Booth F, Richards G, Brown S, Karcher D, Tarlton J. Modeling collisions in laying hens as a tool to identify causative factors for keel bone fractures and means to reduce their occurrence and severity. PLoS One 2018;13:e0200025. doi: 10.1371/journal.pone.0200025 29990363 PMC6038993

[40] Montalcini CM, Petelle MB, Toscano MJ. Commercial laying hens exhibit long-term consistent individual differences and behavioural syndromes in spatial traits. R Soc Open Sci 2023;10. doi: 10.1098/rsos.230043 37234496 PMC10206461

[41] Driver CC, Voelkle MC. Hierarchical continuous time modeling. The Handbook of Personality Dynamics and Processes 2021:887–908. doi: 10.1016/B978-0-12-813995-0.00034-0

[42] Rufener C, Berezowski J, Maximiano Sousa F, Abreu Y, Asher L, Toscano MJ. Finding hens in a haystack: Consistency of movement patterns within and across individual laying hens maintained in large groups. Sci Rep 2018;8. doi: 10.1038/s41598-018-29962-x 30120253 PMC6098140

[43] Hansen I. Behavioural expression of laying hens in aviaries and cages: Frequencies, time budgets and facility utilisation. Http://DxDoiOrg/101080/00071669408417715 2007;35:491–508. doi: 10.1080/00071669408417715 7828008

[44] Odén K, Keeling LJ, Algers B. Behaviour of laying hens in two types of aviary systems on 25 commercial farms in Sweden. Br Poult Sci 2010;43:169–81. doi: 10.1080/00071660120121364 12047079

[45] Campbell DLM, Goodwin SL, Makagon MM, Swanson JC, Siegford JM. Failed landings after laying hen flight in a commercial aviary over two flock cycles. Poult Sci 2016;95:188–97. doi: 10.3382/ps/pev270 26527703

