# Supplementary material for: Keel bone fractures affect laying hens’ mobility, but no evidence for reciprocal effects
Source: PLoS One. 2024 Jul 5;19(7):e0306384. doi: 10.1371/journal.pone.0306384 (PMC11226069; doi:10.1371/journal.pone.0306384)
Supplement: S1 Text — (PDF) [file pone.0306384.s001.pdf]

S1 Text. Datasets description.

The first dataset (Dataset1) used was published by Rufener et al. in 2019 [1], where they tracked 60 Lohmann Brown (LB) and 60 Lohmann Selected Leghorn (LSL) hens using an infrared tracking system. Hens in a pen were not single-strain. All dataset tracked over six days previous to assessing the keel bone. Because the two hybrids exhibited different space-use behaviours, the authors analysed the hybrids separately and suggested the differences could have been caused by a higher susceptibility to stress (due to being part of a phenotypic minority) by LSL hens leading to a more uneven usage of the zones. Therefore, we only included LB hens, with on average 10 (SD = 1.73) observations per hen (4-11 observations / hen, total observations: 593).

We used for the second dataset (Dataset2) data from the group [2], where we tracked 227 Dekalb white hens with a low-frequency tracking system that we previously validated and described in [3]. Raw data were processed via the described ML-method, that uses a classifier trained to detected false registration. Half of the hens hatched-on-farm and the other half hatched in a commercial hatchery. After excluding hens that did not have enough observations for subsequent analysis (i.e. with less than three observations), we were left with 153 hens, mostly because of another study that collected epigenetic samples on focal hens. Hens from this dataset had on average 4 (SD = 0.76) observations (3-5 observations /hen, total observations: 658).

Finally, we used a third dataset (Dataset3) first presented in the current study, where we tracked 169 Dekalb white hens, with the same tracking system as in Dataset2 and processed by filtering all transitions of short duration (less than 1 min) as validated in [3]. We selected randomly half of the focal hens from each pen and relocated them in a new identical pen three times throughout the production period, while the other half staid in their home pen during the entire laying phase. After excluding hens that had less than three observations, we were left with 163 hens (4 hens died, 2 tags were misfunctioning). Hens from this dataset had on average 4 (SD = 0.28) days tracked (3-4 observations /hen, total observations: 638).

References

1. Rufener C, Abreu Y, Asher L, Berezowski JA, Maximiano Sousa F, Stratmann A, Toscano MJ. 2019 Keel bone fractures are associated with individual mobility of laying hens in an aviary system. *Appl Anim Behav Sci* **217**, 48–56. (doi:10.1016/j.applanim.2019.05.007)

2. Montalcini CM, Petelle MB, Toscano MJ. 2023 Commercial hatchery practices have long-lasting effects on laying hens' spatial behaviour and health. *PLoS One* **18**, e0295560. (doi:10.1371/JOURNAL.PONE.0295560)

3. Montalcini CM, Voelkl B, Gómez Y, Gantner M, Toscano MJ. 2022 Evaluation of an Active LF Tracking System and Data Processing Methods for Livestock Precision Farming in the Poultry Sector. *Sensors* **22**, 659. (doi:10.3390/S22020659)
