# Supplementary material for: Keel bone fractures affect laying hens’ mobility, but no evidence for reciprocal effects
Source: PLoS One. 2024 Jul 5;19(7):e0306384. doi: 10.1371/journal.pone.0306384 (PMC11226069; doi:10.1371/journal.pone.0306384)
Supplement: S2 Text — (PDF) [file pone.0306384.s002.pdf]

**S2 Text. Trend and dynamic fluctuation description.**

Each of the four model (one per spatial behaviour) estimated a trend and a dynamic fluctuation around the trend for both the fracture severity and the behaviour. We here give further details pertaining to the trend and the dynamic fluctuations.

## Trend processes

We accounted for the trend of the behaviour and keel bone fracture severity, by incorporating random initial intercepts and random slopes, as well as an estimated auto-effect term for the slopes that varied by process. We allowed varying initial intercepts across hens, by incorporating two latent "**trend" processes** (called here "BHV" and "KBF" in the models) each measured with one manifest indicator (the behaviour and fracture severity) and estimated their initial latent covariance matrix parameters (T0VAR). We estimated their auto-regression effects of time and set their cross-regression effects to 0. To incorporate individual variation in the trend, we specified the continuous intercept parameter as random-effects by incorporating two additional latent **"continuous intercept" processes** (called "cintBHV" and "cintKBF" in the models) influencing the respective original latent processes ("BHV" and "KBF", respectively) with a cross-regression effect of 1 and by estimating the initial latent covariance matrix parameters.

## Dynamics processes

To estimate the dynamic fluctuation around the trend, we incorporated two additional latent **"dynamics" processes**, called "dynBHV" and "dynKBF" in the models. We estimated the auto- and cross-regression effects of these processes (with system noise) to estimate the speed and dynamic of the fluctuation, respectively. We did not allow these two processes to interact or covary with the trend processes and therefore fixed adequate elements from the temporal effects and the initial latent covariances to zero. The dynamics processes, dynBHV and dynKBF, are measured with one manifest indicator, the behaviour and the fracture severity, though account only for the unpredictable change in the observation (i.e. what is not accounted by the trend processes). In other words, the dynamic processes solely estimate the fluctuations around the trend without interacting with the trend.

## Fitted model

We here show the equations of the hierarchical Bayesian continuous time dynamic model used in the present study for the vertical travel distance (i.e., any mention of "BHV" related to the behaviour). The continuous-time temporal cross-effects parameter estimates are contained in the "drift matrix" (off-diagonal), where the direction of the effect should be interpreted from column to row.

Subject parameter distribution:

$$\underbrace{\begin{bmatrix} \text{T0m\_KBF}_i \\ \text{T0m\_BHV}_i \\ \text{T0m\_cintKBF}_i \\ \text{T0m\_cintBHV}_i \end{bmatrix}}_{\boldsymbol{\phi}(i)} \approx \mathrm{N}\left( \begin{bmatrix} -0.9 \\ -0.77 \\ 0.115 \\ 0.255 \end{bmatrix}, \begin{bmatrix} 0.027 & 0.013 & 0.007 & -0.003 \\ 0.013 & 0.399 & 0.022 & -0.18 \\ 0.007 & 0.022 & 0.014 & -0.005 \\ -0.003 & -0.18 & -0.005 & 0.453 \end{bmatrix} \right) + \underbrace{\begin{bmatrix} -0.139 & -0.045 & -0.005 & 0.026 \\ 0.832 & 1.025 & -0.7 & 0.089 \\ 0.367 & -0.151 & 0.068 & 0 \\ -0.747 & -0.169 & -0.223 & 0.017 \end{bmatrix}}_{\boldsymbol{\beta}} \underbrace{\begin{bmatrix} \text{Dataset1} \\ \text{Dataset3} \\ \text{IsOFH} \\ \text{Is\_relocated} \end{bmatrix}}_{\mathbf{z}}$$

Initial latent state:

$$\underbrace{\begin{bmatrix} \text{KBF} \\ \text{BHV} \\ \text{cintKBF} \\ \text{cintBHV} \\ \text{dynKBF} \\ \text{dynBHV} \end{bmatrix}(t_0)}_{\boldsymbol{\eta}(t_0)} \sim \mathrm{N}\left( \underbrace{\begin{bmatrix} -0.9 \\ -0.77 \\ 0.115 \\ 0.255 \\ 0 \\ 0 \end{bmatrix}}_{\underbrace{}_{\text{T0MEANS}}}, \underbrace{UcorSDtoCov\left\{ \begin{bmatrix} 0.016 & 0 & 0 & 0 & 0 & 0 \\ 0.089 & 0.061 & 0 & 0 & 0 & 0 \\ 0.286 & 0.202 & 0.012 & 0 & 0 & 0 \\ 0.006 & -0.338 & 0.002 & 0.066 & 0 & 0 \\ 0 & 0 & 0 & 0 & 0.209 & 0 \\ 0 & 0 & 0 & 0 & 0 & 0.796 \end{bmatrix} \right\}}_{\underbrace{\mathbf{Q^*}_{t0}}_{\text{T0VAR}}} \right)$$

Deterministic change:

$$\underbrace{\mathrm{d}\begin{bmatrix} \text{KBF} \\ \text{BHV} \\ \text{cintKBF} \\ \text{cintBHV} \\ \text{dynKBF} \\ \text{dynBHV} \end{bmatrix}(t)}_{\mathrm{d}\boldsymbol{\eta}(t)} = \left( \underbrace{\begin{bmatrix} -0.217 & 0 & 1 & 0 & 0 & 0 \\ 0 & -1.285 & 0 & 1 & 0 & 0 \\ 0 & 0 & 0 & 0 & 0 & 0 \\ 0 & 0 & 0 & 0 & 0 & 0 \\ 0 & 0 & 0 & 0 & -0.666 & -0.065 \\ 0 & 0 & 0 & 0 & -0.194 & -0.192 \end{bmatrix}}_{\underbrace{\mathbf{A}}_{\text{DRIFT}}} \underbrace{\begin{bmatrix} \text{KBF} \\ \text{BHV} \\ \text{cintKBF} \\ \text{cintBHV} \\ \text{dynKBF} \\ \text{dynBHV} \end{bmatrix}(t)}_{\boldsymbol{\eta}(t)} + \underbrace{\begin{bmatrix} 0 \\ 0 \\ 0 \\ 0 \\ 0 \\ 0 \end{bmatrix}}_{\underbrace{\mathbf{b}}_{\text{CINT}}} \right) \mathrm{d}t \; +$$

Random change:

$$\underbrace{UcorSDtoChol\left\{ \begin{bmatrix} 0 & 0 & 0 & 0 & 0 & 0 \\ 0 & 0 & 0 & 0 & 0 & 0 \\ 0 & 0 & 0 & 0 & 0 & 0 \\ 0 & 0 & 0 & 0 & 0 & 0 \\ 0 & 0 & 0 & 0 & 0.463 & 0 \\ 0 & 0 & 0 & 0 & 0.031 & 0.367 \end{bmatrix} \right\}}_{\underbrace{\mathbf{G}}_{\text{DIFFUSION}}} \underbrace{\mathrm{d}\begin{bmatrix} W_1 \\ W_2 \\ W_3 \\ W_4 \\ W_5 \\ W_6 \end{bmatrix}(t)}_{\mathrm{d}\mathbf{W}(t)}$$

Observations:

$$\underbrace{\begin{bmatrix} \text{severity} \\ \text{VTDperhour} \end{bmatrix}(t)}_{\mathbf{Y}(t)} = \underbrace{\begin{bmatrix} 1 & 0 & 0 & 0 & 1 & 0 \\ 0 & 1 & 0 & 0 & 0 & 1 \end{bmatrix}}_{\underbrace{\boldsymbol{\Lambda}}_{\text{LAMBDA}}} \underbrace{\begin{bmatrix} \text{KBF} \\ \text{BHV} \\ \text{cintKBF} \\ \text{cintBHV} \\ \text{dynKBF} \\ \text{dynBHV} \end{bmatrix}(t)}_{\boldsymbol{\eta}(t)} + \underbrace{\begin{bmatrix} 0 \\ 0 \end{bmatrix}}_{\underbrace{\boldsymbol{\tau}}_{\text{MANIFESTMEANS}}} \; +$$

Observation noise:

$$\underbrace{\begin{bmatrix} 0.294 & 0 \\ 0 & 0.421 \end{bmatrix}}_{\underbrace{\boldsymbol{\Theta}}_{\text{MANIFESTVAR}}} \underbrace{\begin{bmatrix} \epsilon_1 \\ \epsilon_2 \end{bmatrix}(t)}_{\boldsymbol{\epsilon}(t)}$$

System noise distribution per time step: $\Delta\left[W_{j\in[1,6]}\right](t-u) \sim \mathrm{N}(0, t-u)$

Observation noise distribution: $\left[\epsilon_{j\in[1,6]}\right](t) \sim \mathrm{N}(0,1)$

Note: *UcorSDtoChol* converts lower tri matrix of standard deviations and unconstrained correlations to Cholesky factor, *UcorSDtoCov* = transposed cross product of UcorSDtoChol, to give covariance, See Driver & Voelkle (2018) p11.
Individual specific notation (subscript i) only shown for subject parameter distribution – pop. means shown elsewhere.
Linearised approximation of subject parameter distribution shown.
