# Supplementary figures and images for: Keel bone fractures affect laying hens’ mobility, but no evidence for reciprocal effects
Source: PLoS One. 2024 Jul 5;19(7):e0306384. doi: 10.1371/journal.pone.0306384 (PMC11226069; doi:10.1371/journal.pone.0306384)

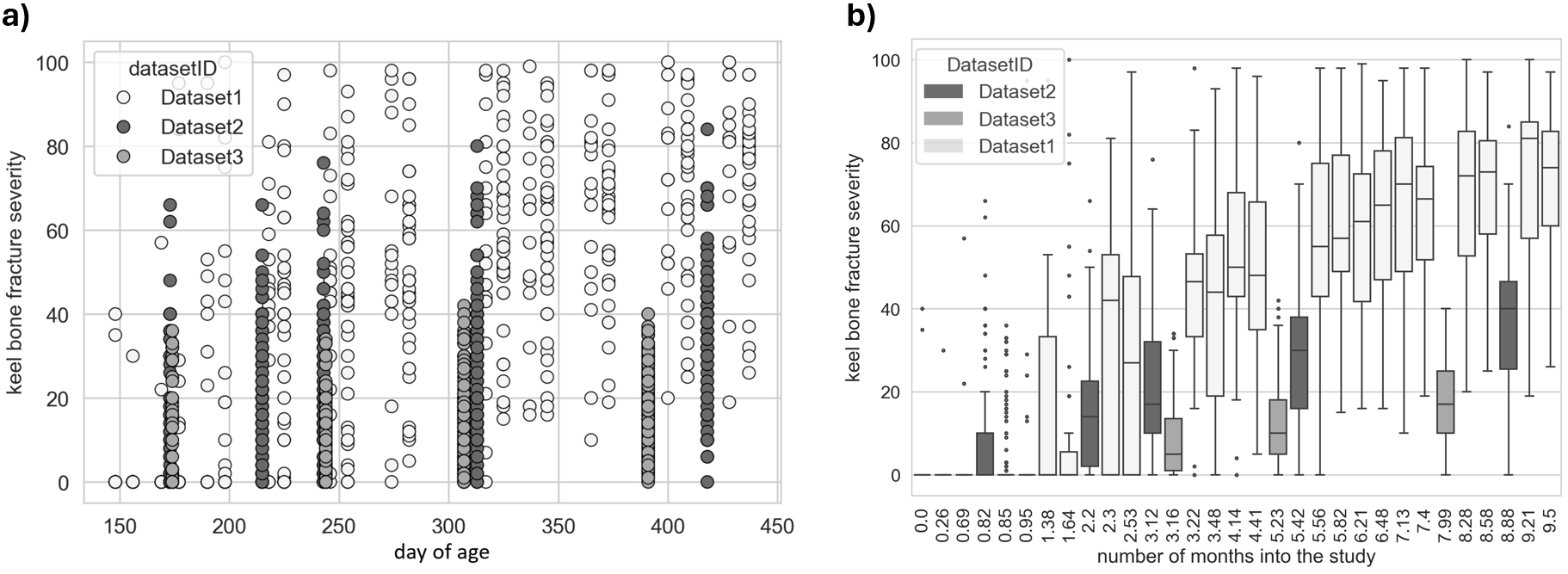

Supplement: S1 Fig — (TIF) [file pone.0306384.s003.tif]

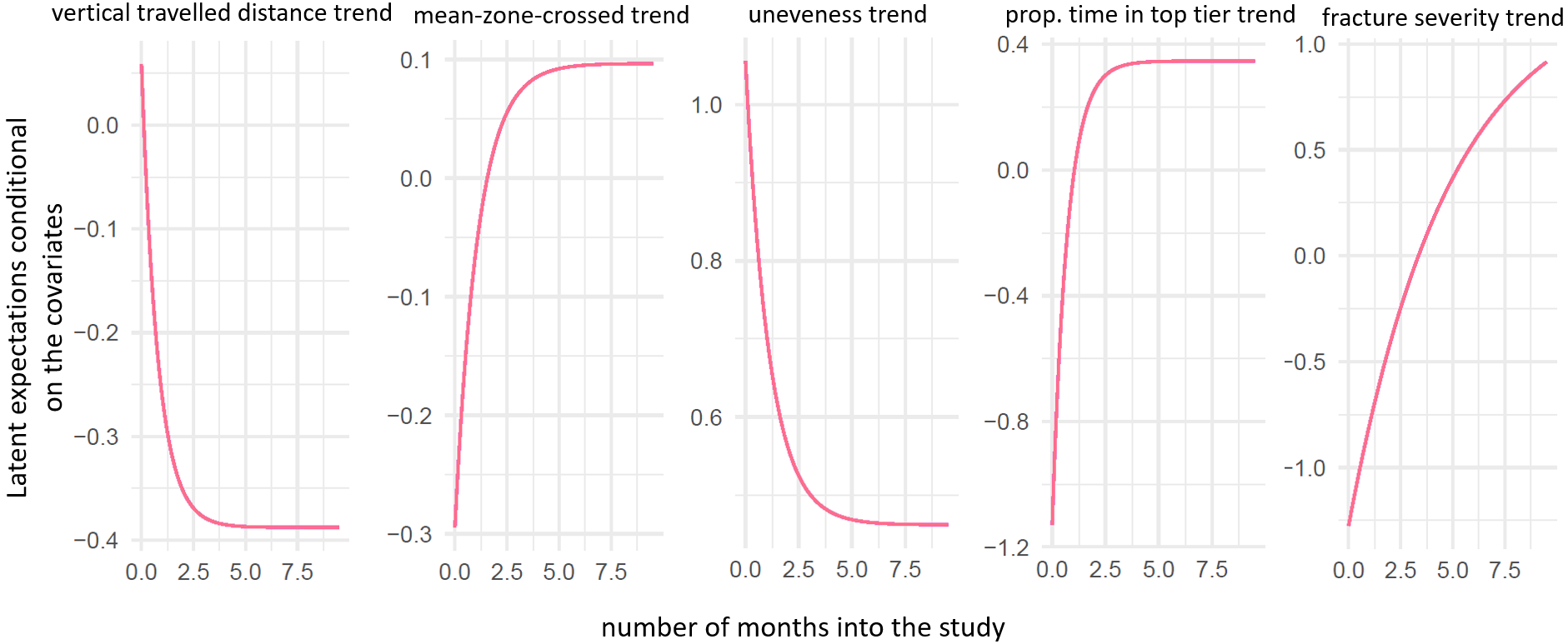

Supplement: S2 Fig — Because the fracture severity trend derived from each model was similar, we solely displayed the one from the model with the mean-zone-crossed. (TIF) [file pone.0306384.s004.tif]
