# Supplementary material for: Keel bone fractures affect laying hens’ mobility, but no evidence for reciprocal effects
Source: PLoS One. 2024 Jul 5;19(7):e0306384. doi: 10.1371/journal.pone.0306384 (PMC11226069; doi:10.1371/journal.pone.0306384)
Supplement: S1 Table — (PDF) [file pone.0306384.s005.pdf]

	vertical travelled distance (/h)	mean-zone-crossed	unevenness	proportion of time spent on the top tier	keel bone fracture severity
Dataset1	3.42 ±2.17	1.04 ±0.07	1.01 ±0.39	0.30 ±0.31	40.34 ±32.97
Dataset2	3.95 ±1.59	1.08 ±0.09	0.78 ±0.36	0.29 ±0.25	21.03 ±18.03
Dataset3	4.11 ±1.51		0.71 ±0.32	0.27 ±0.22	10.21 ±10.40
All datasets	3.84 ±1.79	1.06 ±0.08	0.83 ±0.38	0.29 ±0.26	23.44 ±25.34

S1 Table. Mean and standard deviation of the four spatial behaviours and the keel bone fracture severity across datasets.
